# Assessment of peripheral muscle thickness and architecture in healthy volunteers using hand-held ultrasound devices; a comparison study with standard ultrasound

**DOI:** 10.1186/s12880-019-0373-x

**Published:** 2019-08-19

**Authors:** Peter Turton, Richard Hay, Ingeborg Welters

**Affiliations:** 1 Institute of Aging and Chronic Disease, University of Liverpool, Liverpool, UK; 2 Royal Liverpool University Hospital, Liverpool, UK

**Keywords:** Muscle architecture, Pennation angle, Fascicle length, Muscle ultrasound

## Abstract

**Background:**

Pocket-sized ultrasound devices are increasingly used in a variety of clinical situations, and perform well against standard ultrasound machines. We sought to investigate if a pocket-sized ultrasound device can assess muscle thickness and architecture in healthy volunteers.

**Methods:**

Healthy male volunteers (n = 21) across a range of ages were recruited to the study. Laying supine, ultrasound images were taken from the right anterior and lateral thigh. Thickness of the rectus femoris (RFMT), vastus intermedius (VIMT), and the two combined (anterior thigh, AMT) were measured, along with thickness of vastus lateralis (VLMT), pennation angle (VLPA) and derived fascicle length (VLFL). These scans were performed initially using a pocket-sized ultrasound (VScan) and then using a standard device (Telemed Echoblaster 128).

**Results:**

In all six variables, there was no significant difference between the two sets of measurements. Intra-class correlation co-efficients (ICC) for VLMT, VLPA, and AMT were all excellent (0.93, 0.89, 0.90 respectively) with the derived value of VLFL having an ICC of 0.84. All ICC values were statistically significant. Regression analysis demonstrated no evidence of proportional bias in any of the measured or derived variables.

**Conclusion:**

A pocket-sized ultrasound device gives similar measurements of lower limb muscle thickness and architecture as a standard device in healthy volunteers.

## Background

Pocket-sized ultrasound devices are becoming increasingly common in the clinical setting. The devices are being used in the qualitative assessment of patients, allowing rapid assessment and diagnosis of abdominal [1], aortic [2] and gynaecological [3] pathologies. They have also been shown to be valuable in the assessment of intra-abdominal free fluid in patients with traumatic injuries [4]. Quantitatively, pocket-sized devices have been validated for obstetric measurements [5] and in the estimation of optic nerve sheath diameter [6]. These studies have used the pocket sized Vscan device (GE Healthcare, United States), although other pocket sized devices are described in the literature, such as the Acuson P10 system (Siemens, United States) for use in the assessment of ascites [7].

The use of ultrasound to assess muscle thickness and architecture is well documented across a number of patient populations. For example, ultrasound is used in the critically ill to assess changes in muscle thickness as a result of atrophy [8], and loss of quadriceps muscle has been of interest in patients with respiratory [9], endocrine [10] and renal [11] diseases. In healthy volunteer studies, assessment of muscle architecture with ultrasound has been used to link muscle structure with function [12]. Furthermore, ultrasound systems have been used in sports science to detect fat content within muscle [13], and to estimate depth of subcutaneous fat layers [14].

A recent systematic review concluded that pocket-sized devices produce images that can be used to answer distinct clinical questions, and provide good agreement with high-end devices [15]. The ability to use a pocket-sized device to track changes in muscle size in many patient populations could make muscle assessment easier and cheaper compared to a larger, more expensive device. Similarly, accurate measurement with a pocket-sized device may be of benefit in studies of healthy volunteers. We aim to assess whether a pocket-sized device will accurately measure muscle thickness and architecture in the anterior and lateral thigh, in healthy male volunteers, compared to a standard ultrasound machine.

## Methods

Ethical approval for this study was granted from the University of Liverpool’s central ethics committee. The study was conducted in the gait laboratory within the University’s Institute of Aging and Chronic Disease over a 4 month period, from September to December 2018. This study was performed when participants attended to take part in other studies that involved ultrasound measurements of the thigh muscles. Participants were recruited for these studies through e-mail communications and advertising posters positioned across university notice boards. All recruited participants signed a consent form, having read a participant information sheet and following a verbal briefing from the authors.

### Eligibility criteria

We recruited male participants between the ages of 18 to 70 years old, of any level of physical fitness, and with no significant past medical history. Exclusion criteria were participants outside of the specified age range, a body mass index of greater than 40 kg/m^2^, any history of neuromuscular disorder, previous orthopaedic surgery to either lower limb or recent muscular injury.

### Ultrasound protocol

Participants lay supine on a physiotherapy couch in the anatomical position, with the head supported on one pillow [16]. The right lower limb was used for ultrasound assessment in all participants. Using a marker pen, the placement of the probe for the two anatomical sites of interest was marked; rectus femoris was marked at a point two-thirds of the distance between the anterior-superior iliac spine and the superior tip of the patella on the anterior aspect of the thigh [17] and vastus lateralis was marked at point half-way between the greater trochanter and the popliteal crease [18].

Ultrasound scanning was performed by the same assessor using both probes. The examiner has experience in using ultrasound to assess both muscle thickness and architecture in critically ill patients [19], and has used both devices in a number of heathy volunteer studies. Rectus femoris and vastus lateralis were first imaged using the VScan pocket-sized ultrasound with dual probe (GE Healthcare, United States). The linear probe provides for a 2.9 cm aperture, and works across a frequency range of 3.4 to 8.0 MHz.

For rectus femoris imaging, the depth of imaging was adjusted until the femur was visualised, and the rectus femoris could be seen superior to vastus intermedius. The probe was placed perpendicular to the long axis of the femoral shaft. For vastus lateralis visualisation, the probe was placed parallel to the long axis of the femoral shaft, and the depth of imaging was adjusted until the deep aponeurosis of the vastus lateralis, and the vastus intermedius inferior to it, could be visualised. The probe was tilted to ensure maximal distance between the superficial and deep aponeuroses, and to ensure good visualisation of the fascicles within the muscle body.

A large amount of water-based gel was used, and minimal pressure was placed on the probe to prevent compression of muscle. At each site, three images were taken. The process was then repeated using the linear probe from the Telemed EchoBlaster 128 Ultrasound device (Telemed, Lithuania), which has been used in a number of previous studies to assess peripheral and diaphragm muscle thickness and architecture [20] [21] [22]. A 39 mm linear probe was used at a frequency of 10 MHz.

### Measurements

Images were saved and transferred to a computer, for analysis using ImageJ (NIH, United States) software. Before measurement of the muscle, the measuring scale on each image was itself measured in pixels. The number of pixels on the measuring scale was divided by the length of the scale (in cm) to give the number of pixels per centimetre for that image.

**Fig. 1:**
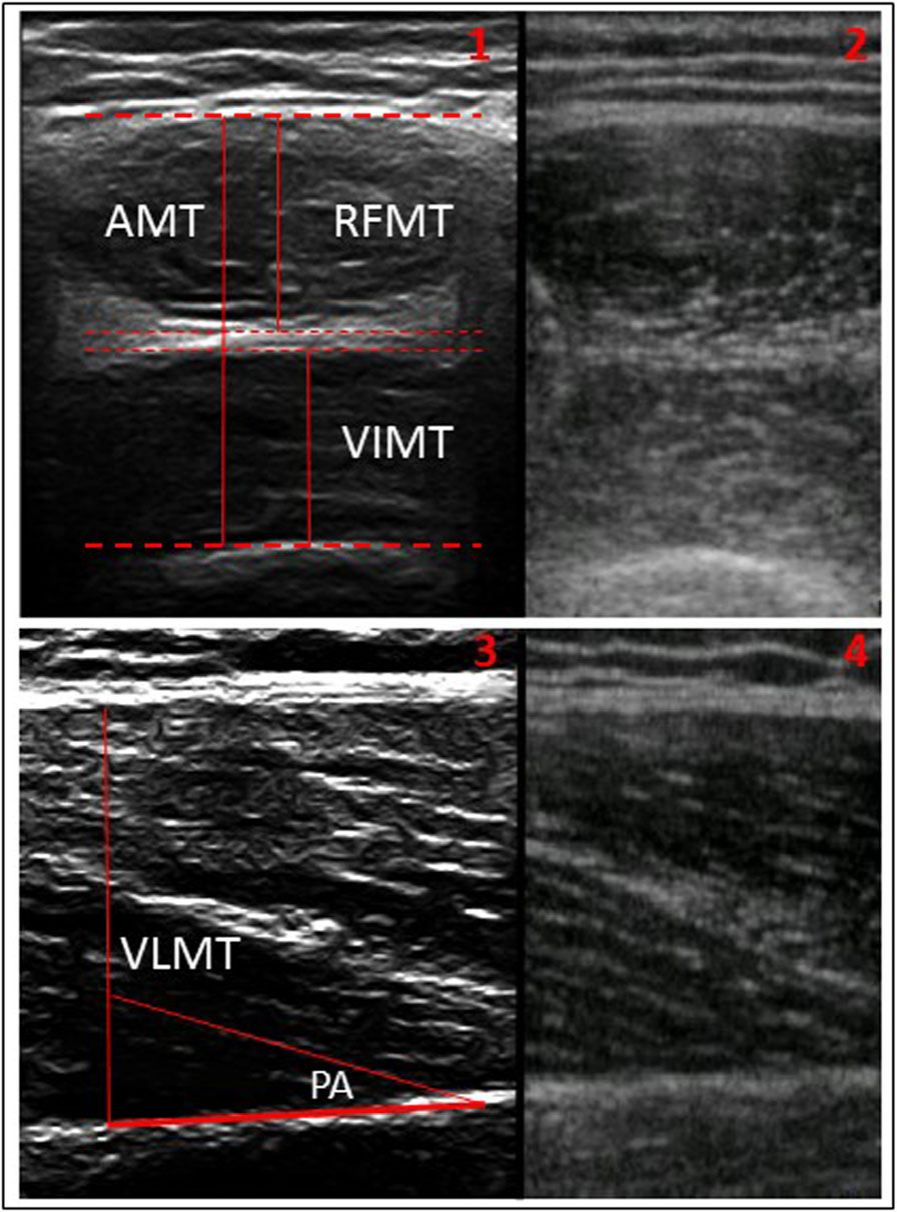
Representative ultrasound scans of the anterior thigh from the Telemed and Vscan systems (panels 1 and 2 respectively) and vastus lateralis (panels 3 and 4 respectively) Legend: AMT: anterior thigh muscle thickness, RFMT: rectus femoris muscle thickness, VIMT: vastest intermedius muscle thickness, VLMT: vastus lateralis muscle thicknes, PA: pennation angle.

For rectus femoris assessment, three measurements were taken. First, rectus femoris muscle thickness (RFMT) was measured as the distance between the inner border of the muscular fascia, down to the hyperechoic interface superior to vastus intermedius. Second, vastus intermedius muscle thickness (VIMT) was measured as the distance from the point most inferior to the hyperechoic interface to the bony surface of the femur. A third combined measurement of anterior thigh muscle thickness (AMT) was measured from the inner border of rectus femoris muscular fascia, down to the bony surface of the femur ( [23], Fig. 1, panels 3 and 4).

For vastus lateralis assessment, two measurements were taken, and a third measurement derived from these two measurements. Muscle thickness (VLMT) was measured at the widest point between the superficial and deep aponeuroses. Pennation angle (VLPA) was measured using the angle-tool, drawing a line connecting the fascicle to the deep aponeurosis, with the angle between this line and the aponeurosis being measured (Fig. 1, panels 3 and 4). Again, this angle was measured at the widest point between the two aponeuroses. Fascicle length (VLFL) was then derived from these two measurements. Assuming that the deep aponeurosis runs at a right angle to the measured line of muscle thickness, the fascicle was treated as the hypotenuse of a right-angled triangle, and calculated from the following formula:

10 $$ VLFL=\mathrm{s} in\ (VLPA)\ x\  VLMT $$

The average measurement from the three images was calculated. Image acquisition and subsequent measurement was performed by one trained investigator using both devices, with the measurement of AMT and VLMT being performed again by a second person in a sample of 11 volunteers, in order to assess the inter-rater agreement.

### Statistical analysis

Comparison of these six measurements was made between the two ultrasound machines by first calculating the difference between the two systems for each measurement, and performing a one-sample t-test for the difference between each system. Intra-class correlation co-efficients (ICC) were calculated, based on a two-way mixed model for absolute agreement between the two systems. For each pair of measurements, the mean measurement was also calculated, to allow creation of Bland-Altman plots with 95% limits of agreement. To detect proportional bias, linear regression was performed, with the mean score between the two systems as the independent variable, and difference between the two systems as the dependent variable.

Statistical testing was performed using SPSS (version 23.0, IBM, United States). In all cases, a p-value of less than 0.05 was considered statistically significant except in the case of proportional bias testing, where a p-value of greater than 0.05 was taken to mean that there was no proportional bias.

## Results

### Sample size and demographics

Twenty-one participants were recruited to this study, and a full set of measurements for each participant was obtained. Demographic data is displayed in Table 1.

**Table 1:** Participant demographics

Variable (n = 21)	Mean value (SD) [Range]
Age (years)	31.67 (14.96) [20–64]
Weight (kg)	81.64 (12.95) [58–114]
Height (metres)	1.80 (0.06) [1.61–1.91]
BMI (kg/m^2^)	25.17 (3.40) [18.94–32.83]

### Validity

Images from eleven participants were double measured by the same person for anterior thigh and vastus lateralis mucle thickness from both ultrasound systems. These measurements were conducted 1 week apart, with an intra-class coefficient (ICC) for consistency of 0.990 and 0.999 (anterior thigh, Telemed and V-scan respectively) and 0.996 and 0.993 (vastus lateralis, Telemed and V-scan respectively). Images from the same 11 participants were then measured by a second blinded observer, giving ICC values for absolute agreement of 0.991 and 0.994 (anterior thigh, Telemed and V-scan respectively), and 0.985 and 0.991 (vastus lateralis, Telemed and V-scan respectively). In all cases, ICC values were significant, with p < 0.001.

### Mean differences and intra-class coefficients (ICC)

**Fig. 2:**
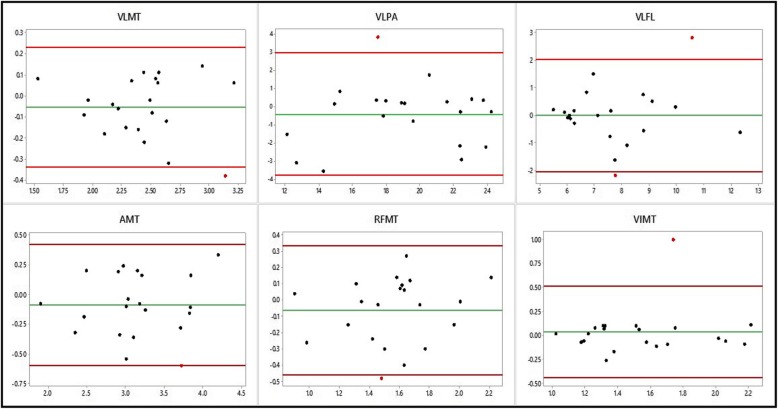
Bland Altman plots for the measured variables. Legend: Green line: mean difference, red lines: 95% limits of agreement, black dots: measurements within the limits of agreement, red dots: measurements outside of the limits of agreement

Intra-class coefficients for the 5 directly measured variables, and the one derived variable, are presented in Table 2. For all 6 variables, the ICC was greater than 0.75, and was statistically significant. The mean differences between the two systems for each measurement were not statistically significant. Bland-Altman plots for all six variables are presented in Fig. 2.

**Table 2:** Mean differences between the two systems and the level of agreement

Variable	Mean difference [SD]	P-value	ICC [95% CI]	P-value
VLMT (cm)	0.05 [0.14]	0.10	0.93 [0.83–0.97]	< 0.001
VLPA (degrees)	0.40 [1.72]	0.29	0.90 [0.77–0.96]	< 0.001
VLFL (cm)	0.01 [1.04]	0.99	0.84 [0.65–0.93]	< 0.001
AMT (cm)	0.09 [0.26]	0.14	0.89 [0.76–0.96]	< 0.001
RFMT (cm)	0.06 [0.20]	0.52	0.80 [0.58–0.91]	< 0.001
VIMT (cm)	−0.03 [0.24]	0.17	0.79 [0.55–0.91]	< 0.001

Where VLMT = vastus lateralis muscle thickness, VLPA = vastus lateralis pennation angle, VLFL = vastus lateralis fascicle length (derived from VLMT and VLPA), AMT = anterior thigh muscle thickness, RFMT = rectus femoris muscle thickness and VIMT = vastus intermedius muscle thickness.

### Proportional bias testing

To assess for proportional bias, linear regression was performed. For each pair of measurements, the mean was calculated by summing the two measurements together and dividing by two, and the difference between the two found by subtracting the measurement with the Vscan system from the measurement with the Telemed system. A regression calculation, with mean measurement as the independent variable and difference as the dependent variable, was calculated. Across all six variables, there was no evidence of proportional bias (see Table 3).

**Table 3:** Regression coefficients for each variable

Variable	Regression co-efficient	P-value
VLMT	0.04	0.60
VLPA	−0.04	0.66
VLFL	−0.06	0.63
AMT	−0.03	0.76
RFMT	−0.11	0.45
VIMT	−0.08	0.64

## Discussion

This study demonstrates that a pocket-sized ultrasound system can be used in healthy volunteers to give accurate assessment of muscle thickness in both the vastus lateralis and the anterior compartment of the quadriceps, compared to a standard ultrasound system. Further, the pocket-sized system can image muscle fascicles to a satisfactory resolution in comparison to a standard system. When fascicle length is determined using trigonometric methods, the resulting length is also comparable to the estimations of fascicle length obtained from the standard device. The lack of proportional bias indicates that this accuracy is maintained across the range of obtained measurements. To our knowledge, this is the first time a pocket-sized device has been used to assess limb muscle architecture, although a different smartphone based pocket-sized ultrasound device has been used successfully in the assessment of the hyomental muscle [24]. The Vscan device used in our study has also been assessed as suitable in the diagnosis of musculoskeletal pathology of the shoulder [25].

The Vscan has been has used in a previous study to assess its accuracy in the measurement of diaphragm thickness at both tidal and maximal end points of respiration [26]. The authors found that measurements of muscle thickness at both inspiration and expiration gave ICC values of greater than 0.9, at both tidal and maximal volumes. They did however find that the system could not accurately assess maximal diaphragm movement, although this is ordinarily measured in M-mode [27], which the Vscan does not possess.

The techniques for assessment of peripheral muscle size and architecture using ultrasound are well established in both healthy volunteers [28] and certain patient populations, with muscle parameters relating to functional outcomes [29]. Muscle architecture specifically describes the arrangement of fascicles within a muscle, with the angle that a fascicle inserts into the deep aponeurosis being known as the pennation angle; this angle is important as it is positively related to force generation, with larger angles being able to pack more muscle into a particular volume [30]. The length of the fascicle spanning between the two aponeuroses indicates the maximal shortening velocity of a muscle [31]. It can be measured directly [32] or estimated using trigonometry if the muscle thickness and pennation angle are known [33]. Rectus femoris cross-sectional area and thickness, vastus intermedius thickness and vastus lateralis thickness have all been shown to significantly correlate with functional measures in patients with critical illness [34], and in healthy volunteers [35], in particular the thickness of vastus intermedius [36].

Our study is limited by the Vscan probe being unable to image the entire cross sectional area of the rectus femoris, possibly due the size of the probe. Although measurement of muscle thickness of the anterior compartment of the thigh is a well-established technique, at present we cannot extrapolate these results to patient populations. For example, in critically ill patients, comparison between thickness and cross sectional area has shown that thickness underestimates loss of muscle by around 8% [37]. Another study has shown that both quadriceps thickness and rectus femoris cross-sectional area decrease significantly in sepsis, but thickness decreases to a lesser degree and does not correlate with volitional measures of strength [38]. Furthermore, the effect of tissue oedema and changes in muscle echogenicity could not be examined in this study of volunteers, and is another limitation in trying to apply these results to any patient population.

Although the absolute agreement across the 6 variables was high, there were reduced ICC values for the measurement of the rectus femoris and vastus intermedius muscles. A possible explanation could be the position and appearance of the hyperechoic interface between the two muscles: themuscular fascia of the rectus femoris is superficial and easily identifiable, as is the echobright tip of the femur, making measurement of the overall anterior compartment of the thigh straightforward [23]. In comparison, the hyperechoic interface between the two muscles that make up the anterior compartment may be harder to clearly delineate using the pre-programmed resolution settings of the Vscan.

Further work in this field should concentrate on the potential benefits of using a pocket-sized devices over a standard ultrasound device. For example, whether a pocket-sized device confers an ergonomic benefit to users, or if the length of time spent acquiring images is similar between the two methods; a simulated study of central venous cannulation found that the time taken to achieve image acquisition and puncture of the vessel was similar between pocket-sized and standard devices [39]. In order to establish a pocket-sized device as a bedside measuring tool, measurements using the device’s own caliper function need to be compared against those obtained using formal computer software, and the use of other commercially available pocket-sized devices could be similarly evaluated for use in assessment of muscle thickness.

Finally, as this was a study performed in healthy volunteers, the device needs to be compared to a standard ultrasound device in patients at risk of muscle wasting, such as those with chronic or critical illness, to assess the effect of oedema and pre-existing sarcopenia on the accuracy of image acquisition.

## Conclusion

In healthy male volunteers, a pocket-sized ultrasound device provides measurements that are comparable to a standard device in the assessment of muscle layer thickness and fascicle architecture. Further work is required to determine if such devices perform as well in patient populations.

## Data Availability

The datasets generated and analysed during the current study are available in the Zenodo repository, www.zenodo.org/record/2649933.

## Abbreviations

AMT: Anterior thigh muscle thickness
ICC: Intra-class correlation coefficient
RFMT: Rectus femoris muscle thickness
VIMT: Vastus intermedius muscle thickness
VLFL: Vastus lateralis fascicle length
VLMT: Vastus lateralis muscle thickness
VLPA: Vastis lateralis pennation angle
